# Use of online and paper-and-pencil questionnaires to assess the distribution of orthorexia nervosa, muscle dysmorphia and eating disorders among university students: can different approaches lead to different results?

**DOI:** 10.1007/s40519-021-01231-3

**Published:** 2021-06-10

**Authors:** Ilaria Silvia Rossella Gorrasi, Cinzia Ferraris, Raffaella Degan, Giovanni Abbate Daga, Simona Bo, Anna Tagliabue, Monica Guglielmetti, Mattia Roppolo, Giorgio Gilli, Daniela Acquadro Maran, Elisabetta Carraro

**Affiliations:** 1 Department of Public Health and Pediatrics, University of Turin, Via Santena 5bis, 10126 Turin, Italy; 2 Department of Neurosciences “Rita Levi Montalcini”, University of Turin, Via Cherasco 11, 10126 Turin, Italy; 3 Department of Medical Sciences, University of Turin, Corso Dogliotti 14, 10126 Turin, Italy; 4 Department of Psychology, University of Turin, Via Verdi 10, 10124 Turin, Italy; 5 Department of Public Health, Experimental and Forensic Medicine, University of Pavia, Via Forlanini 2, 27100 Pavia, Italy

**Keywords:** Orthorexia nervosa, Muscle dysmorphia, Eating disorders, Web-based survey, Paper and pencil survey, Questionnaire

## Abstract

**Purpose:**

Administration of questionnaires to assess the diffusion of disordered eating behaviours via the web is becoming common today. The aim of this study is to assess whether two different approaches of administering a test to assess traits of eating disorders (EDs), orthorexia nervosa (ON) and muscle dysmorphia (MD) by email recruitment and online completion (web-based survey—WBS) and by in person recruitment and paper-and-pencil completion (paper-based survey—PBS), gives different results.

**Methods:**

During 2 consecutive academic years, a self-reported questionnaire consisting of questions about personal characteristics and three tests for the evaluation of ON (ORTO-15), MD (MDDI-ITA), and EDs (EAT-26) were administered to two groups of undergraduates, respectively, as a WBS and a PBS.

**Results:**

The WBS response rate was 6.7% (*N* = 137), and the PBS response rate was 86.5% (*N* = 372). The WBS group showed a statistically significant higher prevalence of students with eating disordered behaviours (21.2% vs 5.4%) and registered a higher mean score on the EAT-26 test (13.5 ± 11.1 vs 6.0 ± 8.0); no differences between the two groups emerged for ON and MD prevalence and test scores. Moreover, in the WBS group, the number of students with one or more tests with test scores above the cut-off values was significantly higher (46.0% vs 32.3%).

**Conclusion:**

The choice of the approach to administer a questionnaire to assess the diffusion of EDs and related issues must take into account all the factors that can result in selection bias and that can affect the reliability of the results.

**Level of evidence:**

Level V, descriptive cross-sectional survey.

**Supplementary Information:**

The online version contains supplementary material available at 10.1007/s40519-021-01231-3.

## Introduction

Several instruments used for psychological and psychiatric clinical and research applications have been validated for administration via the web [1], and this way of administering questionnaires is becoming important in the research field. The preferred mode for collecting survey data in research has traditionally been the paper questionnaire; however, in recent years, this way of collecting data has been challenged [2]. The on-going COVID-19 pandemic for example has highlighted the practical value of using online tools. For the assessment of eating disorders (EDs), since 2013, five new tools have been developed and validated exclusively for online self-report administration or for both online and pencil-and-paper administration [3]. EDs are mental disorders described in the Diagnostic and Statistical Manual of the American Psychiatric Association (DSM-5). Orthorexia nervosa (ON) is not currently recognized as a mental disorder, while muscle dysmorphia (MD) is classified in the DSM-5 as a subtype of body dysmorphic disorder. ON and MD are considered close to EDs [4–9]. The term orthorexia nervosa, literally meaning “proper appetite”, was first coined by Bratman in 1997 [10] to describe an excessive fixation on healthy eating, often associated with significant dietary restrictions and consequent life-threatening medical conditions related to malnutrition, disrupted social life and social isolation. Muscle dysmorphia was first identified by Pope et al. [11] in a group of bodybuilders and refers to individuals preoccupied with their appearance and concerned about not being sufficiently large and muscular, with a life consumed by activities aimed at increasing muscularity, such as weightlifting, dieting and using drugs [12, 13].

The prevalence of ON and MD has been assessed using self-reported questionnaires as screening tools [14–17], administered mostly as paper-and-pencil questionnaires after an in person recruitment [18–25]. More recently, questionnaires have been administered as well online, after a web-based recruitment for example via email or through an advertisement on a website [5, 26–31]. Comparability of the reliability of web-based and paper questionnaires has been supported in some cases [32–34]. It is widely accepted that web-based questionnaires offer advantages, which include more complete data [35], faster return [36, 37], and lower costs [38]. Two main disadvantages have been identified: (1) the relatively high nonresponse rate compared with that from traditional methods and (2) concerns regarding the reliability and validity of the data obtained [39, 40]. Furthermore, when an online test is merely an adaptation of a traditional offline instrument, evidence that the offline version has satisfactory psychometric properties is not sufficient to allow one to assume they will apply to the online version as well [41]. It is important to understand the validity of these measures and why it is necessary to know the accuracy of web *vs* paper-and-pencil questionnaires. For instance, since ON and MD are not currently considered disorders in the DSM-5, it is important to accurately measure ON and MD to assess their prevalence, risk factors, and correlates to examine whether to consider them as clinical disorders or to better understand them as disorders since there is growing awareness that this is how EDs present for many people, especially men.

In the present study, we compare the results of two surveys made to assess the diffusion of ON, MD and ED traits in university students, where two different approaches of administering a questionnaire were used: by email recruitment and online completion (web-based survey—WBS) and by in person recruitment and paper-and-pencil completion (paper-based survey—PBS). The two surveys were conducted at the University of Turin, Italy, during two different and consecutive academic years, enrolling the students attending the first year. The aim of the study was to evaluate if the two different approaches, WBS and PBS, could influence the results of the questionnaire.

## Methods

### Study design and setting

Web-based and paper-based questionnaire surveys were carried out during two different and consecutive academic years, respectively, 2013–2014 and 2014–2015, at the University of Turin.

For the web-based questionnaire survey (WBS), an online questionnaire was developed using the Lime Survey TM (Fa. Carsten Schmitz/Germany). An email invitation (and two reminders), including a link to the website to participate anonymously, were sent to the institutional email addresses of the students. The survey could be completed on all type of devices (computer, smartphone, tablet). The first page of the questionnaire included the information sheet and the informed consent; the affirmative answer to the consent allowed access to the questionnaire. It was divided into four sections, each one organized on several pages based on the number and length of the questions; to move from one page to another it was necessary to click the “next” button. It was mandatory to fill in each question before moving on to the next.

For the paper-and-pencil-based questionnaire survey (PBS), participants were approached during lessons after conferring with professors and were asked to anonymously complete the questionnaire in the classroom. The questionnaire consisted of six pages: the information sheet, the informed consent and the four sections of the questionnaire, one per page.

In both academic years, the survey was presented as an investigation among university students about nutrition habits, approach towards physical activity and proper body aspect. This research was reviewed and approved by the Bioethical Committee of the University of Turin on 01/29/2014.

### Participants

The participants were students attending first year course in medicine, dietetics, physiotherapy, exercise and sport science and business administration, in two different and consecutive academic years. To participate in the survey, it was necessary to give informed consent after taking note of the informative paper.

Students were not compensated for the participation in the study. As an incentive, a personal code was given to each participant: it was included in the email invitation for the WBS and in the informative sheet and in each questionnaire for the PBS. At the end of the study, the codes of the participants with risk traits have been reported on a web page with the indication of the contacts of professionals for supporting students resulted with traits of ON, MD and EDs; in this way, students were able to know their results anonymously and, if desired, to get in touch with the professionals.

### Measures

The questionnaire comprised four sections: (I) questions about personal characteristics and habits as sex, age, weight, height, hours and type of physical exercise, supplements and medicines use, and dieting, (II) the ORTO-15 test [14], which identifies individuals with ON traits, (III) the Muscle-Dysmorphic-Disorder-Inventory Italian version (MDDI-ITA) test [16], which identifies individuals with MD traits, and (IV) the Eating Attitudes Test-26 (EAT-26) [42], which identifies individuals with EDs traits.

### ORTO-15 test

The ORTO-15 test was validated for the Italian population by Donini and colleagues [14]; it is composed of 15 items (for instance: “In the past 3 months, have you felt troubled by the thought of food?”, “Does the thought of food worry you for more than 3 h a day?”) using a four-point Likert scale (always, often, sometimes, never) and participants has to check one answer per item; answers that indicate a risk of ON have a score of “1”, while the “healthier” responses receive a score of “4”. The sum of the points is the final score of the test. Donini and colleagues [14] selected two threshold values below which a diagnosis of at risk of ON could be given: < 40 and < 35, identifying the value of 40 as more predictive of ON. The authors concluded that cut-off point values could be set depending on the purpose for which the scale was used. We chose the cut-off < 35, which showed a high specificity (94.2%) and negative predictive value (91.1%). The value of Cronbach’s alpha coefficient, not reported in the ORTO-15 validation study [14], resulted 0.79 in a later study on Italian athletes [43].

### MDDI-ITA test

MDDI-ITA is a test for the presence of risk of MD. It was validated in the Italian language by Santarnecchi and Dettore [16]; the original English version was developed by Hildebrandt and colleagues [15]. It is composed of 13 items (for example: “I think my legs are too thin”, “I hate my body” or “I feel like I have too much body fat”) rated on a 5-point Likert-type scale (never, rarely, sometimes, often, always) ranging from point “1” for “never” to “5” points for always; participants has to check one answer per item. The sum of the points is the final score of the test. Cronbach’s alpha coefficient was 0.85 [16]. Currently, measurement instruments for MD have not established a defined cut-off score that allows for discrimination of clinically significant results [44]. In this study, we used a cut-off of 39 as previously adopted [18, 28, 45] on the basis of Varangis and colleagues [46], who reported a specificity of 75% and a sensitivity of 73.7%.

### EAT-26 test

EAT-26 is one of the most used tests for identifying subjects with traits of EDs, and it was validated in Italy by Dotti and Lazzari [42]. It is composed of 26 items (always, usually, often, sometimes, rarely, never) and participants have to check 1 answer per item. The sum of the questions yields the total score. The test investigates three different areas of the disorder such as dieting, bulimia and food preoccupation and oral control. For instance, typical questions asked in EAT-26 test are: “I think about food with concern”, “I feel very guilty after eating” or “I feel like throwing up after eating”. The threshold value ≥ 20 identifies subjects at risk of EDs. Cronbach’s alpha coefficient was 0.86 [42].

### Evaluations

The possible differences in the results obtained through the two different approaches of administering the questionnaire, PBS and WBS, were evaluated in terms of: characteristics of participants (sex, age, BMI, hours of physical exercise, supplements and medicines use, dieting); prevalence of traits of ON, MD and EDs; test scores of ORTO-15, MDDI-ITA and EAT-26; students with test scores above the cut-off and with the co-presence of ON, MD and ED traits; correlations between the three test scores, hours of physical activity and BMI.

### Statistical analysis

All data are presented as the mean ± standard deviation or percentage, except for exercise levels that were not normally distributed and are presented as the median (interquartile range). Data were processed using SPSS software, version 25. Data analysis includes descriptive statistics, Student’s *t* test or a *χ*2 test to assess the significant differences in variables between the two groups (e.g., BMI or students with ON traits and students without ON traits), Mann–Whitney test to compare physical exercise levels, alpha reliabilities (*α*—Cronbach’s alpha) for each scale and Pearson-*r* for correlation analyses between the test scores, physical activity levels and BMI.

## Results

### Participants

For the WBS, an email invitation was sent to 2047 students: 180 (8.8%) entered the web page of the questionnaire, 18 did not give their informed consent, 162 agreed to participate and 137 completed the questionnaire, with a response rate of 6.7%.

For the PBS, the questionnaire was administered to 430 students: 372 provided a complete questionnaire response, with a response rate of 86.5%. The rest of the students did not complete all the questions thus these questionnaires were not considered in the statistical analysis.

### Sample characteristics and questionnaire results

The descriptive characteristics of the sample are presented in Table 1. Considering personal characteristics and habits, the group of students who filled out the paper-based questionnaire is engaged in more physical activity than the group who filled out the web-based questionnaire. Regarding the distribution of the participants in the degree courses, no difference in the distribution of medicine students between the two groups has been found, while the students of dietetics and physiotherapy and of exercise and sport science were more represented (*p* = 0.044) and those of business administration were less represented (*p* = 0.002) in the PBS group. Making a comparison between the WBS and the PBS groups, no statistically significant differences emerged by sex, BMI, supplements and medicines use or dieting. However, a statistically significant higher weekly physical activity in PBS group than in WBS was observed, probably due to the greater number of students of exercise and sport science course in the PBS group.

**Table 1:** Characteristics of participants

	Web-based questionnaire survey (*n* = 137)	Paper-based questionnaire survey (*n* = 372)	*p*
Males, *n* (%)	53 (38.7)	160 (43.0)	0.380^a^
Age (years)	20.4 ± 2.8	20.0 ± 1.3	0.082^b^
Medicine course, *n* (%)	69 (50.4)	183 (49.2)	0.814^a^
Dietetics and physiotherapy course, *n* (%)	7 (5.1)	36 (9.7)	0.044*^a^
Exercise and sport science course, *n* (%)	24 (17.5)	97 (26.1)	0.044*^a^
Business administration course, *n* (%)	37 (27.0)	56 (15.0)	0.002*^a^
BMI (kg/m^2^)	21.4 ± 2.8	22.1 ± 2.8	0.824^b^
Physical activity (h/week)	4.0 ± 4.0	4.5 ± 7.5	0.020*^c^
Supplement use, *n* (%)	14 (10.2)	60 (16.1)	0.093^a^
Medicine use, *n* (%)	15 (10.9)	42 (11.3)	0.914^a^
Dieting, *n* (%)	16 (11.7)	31 (8.3)	0.248^a^
Traits of ORTO-15 cut-off < 35, *n* (%)	49 (35.8)	105 (28.2)	0.100^a^
Traits of MD cut-off > 39, *n* (%)	10 (7.3)	21 (5.6)	0.489^a^
Traits of EDs cut-off ≥ 20, *n* (%)	29 (21.2)	20 (5.4)	< 0.001*^a^

*Statistically significant *p* values

^a^*χ*2 test

^b^Student’s *t* test

^c^Kruskal–Wallis test

Analysing the results of the three tests that evaluated ON, MD and EDs, a statistically significant difference emerged only for EDs: the WBS group showed a higher prevalence and a higher score with the EAT-26 scale than the PBS group did, while no differences between the two student groups emerged in terms of prevalence of ON and MD traits or in terms of ORTO-15 and MDDI-ITA test scores (Tables 1, 2). As shown in Table 2, the Cronbach’s alpha values for ORTO-15 test in both groups resulted lower than that obtained in a previous study (*α* = 0.79) [43]; moreover, the Cohen’s *d* value reveals an effect due to the size differences between the WBS and PBS groups for the EAT-26 test.

**Table 2:** Results of the three tests (means score value ± s.d.) obtained in the WBS and PBS groups

	Web-based questionnaire survey *n* = 137	Paper-based questionnaire survey *n* = 372	*t* test *p*	Cohen’s *d*
Score ORTO-15	35.7 ± 3.8 (*α* = 0.305)	36.5 ± 3.9 (*α* = 0.312)	0.819	0.20
Score MDDI-ITA	28.0 ± 7.3 (*α* = 0.727)	26.1 ± 7.3 (*α* = 0.747)	0.922	0.26
Score EAT-26	13.5 ± 11.1 (*α* = 0.895)	6.0 ± 8.0 (*α* = 0.874)	< 0.001*	0.84

*α* Cronbach’s alpha value

*Statistically significant *p* values

Due to the internal consistence of ORTO-15, all the results must be interpreted with caution. As suggested by Meule and colleagues [47], we tried to reverse the score in some items (for example item 8). However, the result of Cronbach’s alpha did not change. According to the authors mentioned above, the ORTO-15 has problems in wording and scoring and a revision of the Italian version is needed.

Because of the extreme difference in number of students belonging to the different degree courses, it was impossible to make a comparison between the WBS and PBS groups of the results obtained with the three tests in the different degree courses.

### Students with test scores above the cut-off and with a co-presence ON, MD and ED traits

The co-presence of a risk factor for two or three simultaneous conditions was registered in both groups. For the two groups, the analysis of the distribution of the number of students who had none, one or more test scores above the cut-off values in the three submitted test responses (ORTO-15, MDDI-ITA and EAT-26) showed a statistically significant difference: the number of students without any risk condition was higher in the group that filled out the paper-based questionnaire than that in the web-based questionnaire group, and in the same group, the number of students with one or more test scores above the cut-off values was lower than the number of web-based questionnaire students (Table 3).

**Table 3:** Number of students with test scores above the cut-off

Number of tests with scores above the cut-off values	Web-based questionnaire survey (*n* = 137)	Paper-based questionnaire survey (*n* = 372)	*p*
0 test, *n* (%)	74 (54.0)	252 (67.7)	0.002*
1 test, *n* (%)	43 (31.4)	100 (26.9)	
2 test, *n* (%)	15 (10.9)	14 (3.8)	
3 test, *n* (%)	5 (3.6)	6 (1.6)	

*Statistically significant *p* values

### Correlations among the three test scores, hours of physical activity and BMI

In the WBS and PBS groups, ON, MD and ED were correlated (Pearson correlation) with each other. In both groups of students, the ORTO-15 scores were negatively correlated with the MDDI-ITA and EAT-26 scores; a lower score on the ORTO-15 test corresponded to a greater attitude towards ON: the correlations found suggest that as the orthorexic attitudes increase, attitudes for behaviours typical of MD or EDs also increase. MDDI-ITA scores were correlated with EAT-26 scores and with hours of physical activity. Moreover, only in the PBS group, MDDI-ITA was also correlated with BMI, and the EAT-26 was negatively correlated with hours of physical activity (Table 4).

**Table 4:** Correlations among test scores, BMI and physical activity

	Web-based questionnaire survey (*n* = 137)			Paper-based questionnaire survey (*n* = 372)		
	ORTO-15	MDDI-ITA	EAT-26	ORTO-15	MDDI-ITA	EAT-26
Physical activity (h/week), *r*	− 0.104 (0.225)	0.152*(0.038)	− 0.11 (0.902)	− 0.037 (0.478)	0.117*(< 0.001)	− 0.141* (0.006)
BMI, *r* (sig.)	− 0.017 (0.841)	0.152 (0.077)	− 0.013 (0.876)	0.084 (0.108)	0.172* (0.001)	− 0.024 (0.647)
ORTO-15, *r* (sig.)	–	− 0.283* (0.001)	− 0.362* (< 0.001)	–	− 0.269*(< 0.001)	− 0.307* (< 0.001)
MDDI-ITA, *r* (sig.)	–	–	0.594* (< 0.001)	–	–	0.420*(< 0.001)

*r* Spearman’s rank correlation coefficient

*Statistically significant *p* values

## Discussion

In this study, we assessed the comparability of the results obtained from two surveys carried out among students at the University of Turin to evaluate the prevalence of ON, MD and ED traits using different approaches of administering the questionnaire: by email recruitment and online completion (web-based survey—WBS) and by in person recruitment and paper-and-pencil completion (paper-based survey—PBS). Surveys were carried out in two consecutive academic years and involved students attending their first year in degree courses of medicine, dietetics, physiotherapy, exercise and sport science and business administration; the number of students enrolled in university courses in the two academic years was comparable.

In the web-based questionnaire survey (WBS), 2047 students were invited to participate via email, and the response rate was very low, 6.7%, with only 162 acceptances and 137 questionnaires completed. In the paper-and-pencil-based questionnaire survey (PBS), the number of students approached and invited to participate in the survey was lower, totalling 430 students, however, the response rate was 86.5%. But all agreed to participate (100%), and the response rate was 86.5%. The shift from WBS to PBS was decided, because the response rate with WBS was very low. There were two reasons why fewer students were approached during lessons than were invited via email: (1) courses with a large number of students enrolled (i.e., business administration and exercise and sport science) divide students into more than one class, and we did not have the personnel or sufficient time to administer questionnaires in all the classes; and (2) since mandatory attendance is not required for all university courses, it is difficult to reach all students during lessons. About personal characteristics and habits, comparing the descriptive characteristics of the WBS and PBS groups, the only significant difference was a lesser amount of hours per week of physical activity in the WBS group, that is probably due to the presence of a higher number of students of Exercise and sport science course in the PBS group: the students of dietetics and physiotherapy and of exercise and sport science in fact were more represented and those of business administration were less represented.

Analysing the results of the three tests no significant differences have been revealed between WBS and PBS groups with ON and MD scales; however, the WBS group presented significantly higher scores with the EAT-26 test and a greater number of subjects with ED traits than the PBS group. Furthermore, in the WBS group, the number of students with tests with scores above the cut-off values and the number of students with one or more tests with score above the cut-off was significantly greater. While taking into account the limitation due to the different sizes of the two groups for EAT-26 test (Cohens’ *d* value = 0.84), these results indicate that in the WBS group, there was a major prevalence of EDs traits, and more generally, considering the number of tests with scores above the cut-off values, there were more students with traits for the conditions examined than in the PBS group. Taking into account the low response rate obtained in the WBS group, the finding of a great number of subjects with critical traits towards eating disorders and the other problems investigated in this group could be due to a selection bias of the WBS participants caused by a greater propensity of the subjects with these traits to fill out the questionnaire.

Analysing the validity of the results obtained with the three tests, another important issue is associated to the choice of ORTO-15 test for ON evaluation, which reliability measured by Cronbach’s alpha resulted low with both administration methods (WBS *α* = 0.305; PBS *α* = 0.312). The publication in which the ORTO-15 was validated dates back to 2005 and on that occasion, the value of Cronbach’s alpha was not reported. The reliability of the scale was measured in some subsequent articles, where the test was applied in groups of subjects with particular characteristics (athletes and patients with eating disorders) obtaining alpha values towards 0.79–0.81 [43, 48]. Unfortunately, the poor reliability of this test encountered in our study is in line with other studies [49], where authors tried to validate the test in other countries [47].

According to Meule and colleagues [47], ORTO-15 has problems in wording and scoring and a revision of the Italian version is needed. The review of this test is currently underway [50, 51], also in relation to the changes in the food approach and lifestyles of the Italian population that have occurred since 2005 to date. About the results of our investigation, the alpha values lead to consider the results obtained from ORTO-15 with caution.

It is known that web-based administration may yield slightly different results compared with those obtained from paper-and-pencil assessments [52, 53], and it has been documented that the mode of test administration affects the expected score distributions [41]. Moreover, web-based questionnaires have been concerned about the reliability and validity of the data obtained. Studies in various areas of health research have shown that traditional epidemiologic risk factors, such as perceived health status, anthropometry data, and smoking and alcohol use, can be collected with equal or even better reliability in web-based questionnaires than with traditional approaches [39, 54].

In our study, we attribute the differences of the test scores between online and paper-and-pencil administration to three main aspects: the low response rate in the WBS and a possible resulting selection bias, the absence of a validation of the online versions of the three tests, and a different approach of participants towards online questionnaires *vs* paper-and-pencil questionnaires.

Low response rates represent a major concern that threatens the quality of the web surveys [55], and self-selection is a common cause of selection bias [39]. Also, traditional modes of data-collection are facing a drop in response rates, and concerns about nonresponse bias are not only applicable to online data collection [39, 56–58]. Actual data on web surveys yielded, on average, a 12% points lower response rate compared with other survey modes (range from 1.4 to 82.1% response rate) [59, 60]; the response rate obtained in our study and in other studies using online assessments of ON and EDs are generally in the same range [30, 61, 62]. A rate response of 18.4% was obtained from 11,828 students at two universities in the USA after sending an email invitation to complete the EDE-Q test for assessing the prevalence of EDs [61]. Tremelling and colleagues [62] obtained a response rate of 27.4% among a sample of 2500 dietitian nutritionists invited via email to complete the ORTO-15 and EDE-Q tests in Texas, concluding that choosing to participate or not could influence results regarding the presence of ON traits among respondents [62]. Dell’Osso and colleagues [30] sent an email invitation to the whole student population at the University of Pisa, Italy, (51,609 subjects) to fill out the ORTO-15, and the response rate was 4.13%, a factor that reduced the generalizability of the study results according to the authors. A higher rate was obtained by Parra-Fernandez and colleagues [31]: on 640 university students who were asked to complete an online questionnaire through the JotForm platform, they had a response rate of 70.28%.

Among the factors that can influence the response rate in web surveys there are the sponsors, the topic, the length of time required to complete the survey, the presentation of the questionnaire, the contact delivery modes, the use of pre-notifications and the presence of incentives [55]. According to a review of web surveys [55], several meta-analyses have shown that the salience of a topic is one of the most important factors that influences the response rates to both mail and web surveys [63–65]. When the topic is of high salience (i.e., the topic is of high interest to some surveys), potential respondents are more likely to respond to the survey [55].

Therefore, we could hypothesize that in our study, the topic of the survey presented through the WBS attracted a high number of subjects interested in issues related to nutrition, body aspect and physical activity. In contrast, in the PBS, all students approached in class agreed to participate, reducing the self-selection bias of the sample.

To our knowledge, there is no validation of the online version of MDDI-ITA or of the EAT-26 test. The validity of an online version of the ORTO-15 translated into Portuguese was tested among a sample of Brazilian dieticians, but no evidence was found of its validity and reliability with the initial psychometric evaluation performed [66]. Some studies used online adaptations of ORTO-15 [26, 27, 30, 62, 67, 68], MDDI-ITA [28] or its original English version [69] and EAT-26 [5, 69–71]. While most of the evidence to date indicates that online adaptations of offline tests usually address the expected constructs, there have been sufficient indications of (usually small) differences (e.g., in factor structure, score distributions) to advocate caution, especially in instances where test use has real implications for people’s well-being [41]. According to Buchanan, when an online test is an adaptation of a traditional offline instrument, evidence that the offline version has satisfactory psychometric properties is not sufficient to allow one to assume they will apply to the online version as well [41]. In our study, the use of tools not even validated for psychometric properties for online administration can be a limitation in the results obtained.

A strong candidate for explaining the reasons behind the differences in the scores between online and paper-and-pencil surveys is increased self-disclosure [1]. There is compelling evidence that people may disclose more about themselves when communicating via computers than via face-to-face interactions [72], a phenomenon that appears to extend to internet-mediated communication [73]. This has actually been one of the possible advantages suggested for online clinical work and has also led to the suggestion that online psychological questionnaires will actually give a better picture of the individual’s real personality than traditional measures would [1]. Electronic administration of questionnaires can affect the responses given to self-administered survey questionnaires through direct influence on the respondents [74]. For example, concerns about privacy, anonymity and confidentiality might influence the accuracy of the answers to certain items, and social and cultural beliefs can influence the acceptability of the response [74, 75]. In the PBS, the students completed the questionnaire in the classroom; this aspect may have affected the self-disclosure, as students were not alone during the completion but with their classmates.

A correlation among the three test scores was identified in both the WBS and PBS groups: the increase in attitudes towards one of the conditions examined also implies an increase in attitudes towards the other two conditions. This is in line with the literature: a correlation between ON and ED traits emerged in some studies [6, 67, 76, 77] as well as a correlation between MD and EDs [78–80]. This result is also in line with the studies in which a proximity of features between ON and MD with EDs is considered [4–9, 25].

The use of electronic self-administered survey questionnaires has become common in several research areas [81]. In situations such as the current COVID-19 pandemic, the importance of using online tools emerges more than ever. It is, therefore, essential that these tools are valid and representative and it is important to consider the impact that changing the mode of delivery can have on the responses collected [74]. In this study, the same questionnaire for evaluating the prevalence of ON, MD and ED traits administered in 2 consecutive years to an analogous group of undergraduates online and via paper-and-pencil gave some different results: in comparison with the PBS group students who filled the online questionnaire had a higher prevalence of traits for EDs and a higher number of subjects with traits for one or more of the three conditions examined. These differences could be due to an effective distinction between the two groups, but the size differences between the two groups and the different approach for the questionnaire administration can also play a significant role. It is important to address correctly online surveys, preferably requiring instruments specifically validated for that use.

This investigation has some limitation. The first is related to the different numbers of students participating at the surveys in the two groups (*n* = 137 in the WBS group; *n* = 372 in the PBS group) that could have affected some results, as for EAT-26 (Cohen’s *d* value = 0.84) and the particularly low response rate obtained with the WBS (6.7%) could be another problem concerning the interpretation of the results obtained with the three tests. Another limitation is about the social desirability responding (the tendency to reply to questionnaire giving a more favorable image of him/her-self [82]. About the scales, as discussed above, the ORTO-15 needs a revision in wording and scoring: the internal consistence of this scale highlights the limits of the Italian version of this measure. Moreover, the two study cohorts used to obtain online and paper–pencil show differences regarding type of study and physical activity: these variables can be included as covariates in further analyses. Finally, for the limited information we gathered on this subject, we could not control the effect of socio-economic variables on the scores obtained from paper–pencil and online assessments, and therefore, correct the results for the background characteristics of participants.

Despite these limitations, results from this investigation could help scholars to choose the different type of administration of ORTO-15, MDDI-ITA and EAT-26 questionnaire: findings show that WBS, in particular, permits a larger participation than PBS.

## What is already know on the subject?

Online surveys to assess the diffusion of eating disorders, both classified and emerging as orthorexia nervosa and muscle dysmorphia have been widely used in recent years, however, the questionnaires used are not generally validated for online administration. The results of online surveys may be affected by bias due for example to low response rates, to a self-selection linked to the salience of a topic, the sponsors, the length of time required to complete the survey, the presentation of the questionnaire, the contact delivery modes, the use of pre-notifications and the presence of incentives. Furthermore, in online surveys, subjects often have greater self-disclosure. The results of the web-based surveys must take into account all these aspects to be considered valid and reliable.

## What your study adds?

This study, for the first time to our knowledge, compares the results obtained with the online and paper administration of questionnaires for the evaluation of the diffusion of EDs, ON and MD on analogous groups of university students. Differences between the groups have been identified. The results highlight the need for an adequate design of web-based surveys and the importance of using validated questionnaires for this type of administration.

## Supplementary Information

Below is the link to the electronic supplementary material.

Supplementary file1 (DOCX 271 kb)

## Data Availability

The dataset generated and analysed during the current study are available from the corresponding author on reasonable request.
