# Supplementary material for: Use of online and paper-and-pencil questionnaires to assess the distribution of orthorexia nervosa, muscle dysmorphia and eating disorders among university students: can different approaches lead to different results?
Source: Eat Weight Disord. 2021 Jun 10;27(3):989–99. doi: 10.1007/s40519-021-01231-3 (PMC8190766; doi:10.1007/s40519-021-01231-3)
Supplement: Supplementary file 1 — Supplementary file1 (DOCX 271 kb) [file 40519_2021_1231_MOESM1_ESM.docx]

**Test ORTO-15**

Translated in Italian from “Donini LM, Marsili D, Graziani MP, et al (2005) Orthorexia nervosa: Validation of a diagnosis questionnaire. Eat Weight Disord - Stud Anorexia, Bulim Obes 10:e28–e32. <https://doi.org/10.1007/BF03327537>”

|  | **Sempre** | **Spesso** | **Qualche**  **volta** | **Mai** |
| --- | --- | --- | --- | --- |
| 1. Quando mangi, presti attenzione alle calorie del cibo? | □ | □ | □ | □ |
| 2. Ti senti confuso quando ti trovi in un negozio di generi alimentari? | □ | □ | □ | □ |
| 3. Negli ultimi tre mesi, ti sei sentito preoccupato dal pensiero del cibo? | □ | □ | □ | □ |
| 4. Le tue scelte alimentari sono condizionate dalla preoccupazione per il tuo stato di salute? | □ | □ | □ | □ |
| 5. Il gusto del cibo è più importante della qualità, quando? | □ | □ | □ | □ |
| 6. Sei disposto a spendere molto denaro per avere un cibo salutare? | □ | □ | □ | □ |
| 7. Il pensiero del cibo ti preoccupa per più di 3 ore al giorno? | □ | □ | □ | □ |
| 8. Ti capita di trasgredire con l’alimentazione? | □ | □ | □ | □ |
| 9. Pensi che il tuo umore possa incidere sulle tue abitudini alimentari? | □ | □ | □ | □ |
| 10. Pensi che mangiare cibi più sani possa aumentare la tua autostima? | □ | □ | □ | □ |
| 11. Pensi che mangiare cibi sani possa comportare un cambiamento nel tuo stile di vita (frequenza dei pasti fuori casa, amicizie, …)? | □ | □ | □ | □ |
| 12. Pensa che mangiare cibi salutari possa migliorare il tuo aspetto? | □ | □ | □ | □ |
| 13. Ti senti colpevole quando trasgredisci? | □ | □ | □ | □ |
| 14. Pensi che nei supermercati si possa trovare anche cibo poco sano? | □ | □ | □ | □ |
| 15. Attualmente, consumi da solo i tuoi pasti? | □ | □ | □ | □ |

**MDDI-ITA test (Muscle Dysmorphia Disorder Inventory Test – Italian version)**

Santarnecchi E, Dèttore D (2012) Muscle dysmorphia in different degrees of bodybuilding activities: Validation of the Italian version of Muscle Dysmorphia Disorder Inventory and Bodybuilder Image Grid. Body Image 9:396–403. https://doi.org/10.1016/j.bodyim.2012.03.006

**EAT 26 - Eating Attitude Test 26**

Dotti A, Lazzari R (1998) Validation and reliability of the Italian EAT-26. Eat Weight Disord - Stud Anorexia, Bulim Obes 3:188–194. <https://doi.org/10.1007/BF03340009>

|  | **Sempre** | **Molto spesso** | **Spesso** | **Qualche**  **volta** | **Raramente** | **Mai** |
| --- | --- | --- | --- | --- | --- | --- |
| 1. Ho una terribile paura di ingrassare | □ | □ | □ | □ | □ | □ |
| 2. Quando ho fame evito di mangiare | □ | □ | □ | □ | □ | □ |
| 3. Penso al cibo con preoccupazione | □ | □ | □ | □ | □ | □ |
| 4. Mi è capitato di mangiare con enorme voracità sentendomi incapace di smettere | □ | □ | □ | □ | □ | □ |
| 5. Ho l’abitudine di sminuzzare il cibo | □ | □ | □ | □ | □ | □ |
| 6. Faccio molta attenzione al potere calorico dei cibi che mangio | □ | □ | □ | □ | □ | □ |
| 7. Evito in particolare i cibi con elevato contenuto di carboidrati (pasta, pane,dolci) | □ | □ | □ | □ | □ | □ |
| 8. Sento che gli altri vorrebbero che io mangiassi di più | □ | □ | □ | □ | □ | □ |
| 9. Mi capita di vomitare dopo aver mangiato | □ | □ | □ | □ | □ | □ |
| 10. Mi sento molto in colpa dopo aver mangiato | □ | □ | □ | □ | □ | □ |
| 11. Mi tormenta il desiderio di essere più sottile | □ | □ | □ | □ | □ | □ |
| 12. Mentre faccio sport penso alle calorie che sto bruciando | □ | □ | □ | □ | □ | □ |
| 13. Gli altri pensano che io sia troppo magro/a | □ | □ | □ | □ | □ | □ |
| 14. Mi preoccupa l’idea di avere del grasso sul corpo | □ | □ | □ | □ | □ | □ |
| 15. Impiego più tempo degli altri per mangiare | □ | □ | □ | □ | □ | □ |
| 16. Evito cibi dolci | □ | □ | □ | □ | □ | □ |
| 17. Mangio cibi dietetici | □ | □ | □ | □ | □ | □ |
| 18. Sento che il cibo domina la mia vita | □ | □ | □ | □ | □ | □ |
| 19. Mi piace mostrare un grande autocontrollo verso il cibo e dominare la fame | □ | □ | □ | □ | □ | □ |
| 20. Sento che gli altri fanno pressioni su di me perché io mangi | □ | □ | □ | □ | □ | □ |
| 21. Dedico al cibo troppo tempo e troppi pensieri | □ | □ | □ | □ | □ | □ |
| 22. Mi dispero se mangio dei dolci | □ | □ | □ | □ | □ | □ |
| 23. Mi piace che il mio stomaco sia vuoto | □ | □ | □ | □ | □ | □ |
| 24. Mi impegno in programmi di dieta | □ | □ | □ | □ | □ | □ |
| 25. Mi piace provare nuovi cibi elaborati | □ | □ | □ | □ | □ | □ |
| 26. Ho l’impulso a vomitare dopo mangiato | □ | □ | □ | □ | □ | □ |

**ORTO-15 test**

Donini LM, Marsili D, Graziani MP, et al (2005) Orthorexia nervosa: Validation of a diagnosis questionnaire. Eat Weight Disord - Stud Anorexia, Bulim Obes 10:e28–e32. <https://doi.org/10.1007/BF03327537>

**MDDI - Muscle Dysmorphia Disorder Inventory Test**

Santarnecchi E, Dèttore D (2012) Muscle dysmorphia in different degrees of bodybuilding activities: Validation of the Italian version of Muscle Dysmorphia Disorder Inventory and Bodybuilder Image Grid. Body Image 9:396–403. <https://doi.org/10.1016/j.bodyim.2012.03.006>

|  | **Never** | **Rarely** | **Sometimes** | **Often** | **Always** |
| --- | --- | --- | --- | --- | --- |
| 1. I think my body is too small | □ | □ | □ | □ | □ |
| 2. I wear loose clothing so that  people cannot see my body | □ | □ | □ | □ | □ |
| 3.I hate my body | □ | □ | □ | □ | □ |
| 4. I wish I could get bigger | □ | □ | □ | □ | □ |
| 5. I think my chest is too small | □ | □ | □ | □ | □ |
| 6. I think my legs are too thin | □ | □ | □ | □ | □ |
| 7. I feel like I have too much body fat | □ | □ | □ | □ | □ |
| 8. I wish my arms were bigger | □ | □ | □ | □ | □ |
| 9. I am very shy about letting  people see me with my shirt off | □ | □ | □ | □ | □ |
| 10. I feel anxious when I miss  one or more workout days | □ | □ | □ | □ | □ |
| 11. I pass up social activities  with friends because of my workout schedule | □ | □ | □ | □ | □ |
| 12. I feel depressed when I miss  one or more workout days | □ | □ | □ | □ | □ |
| 13. I pass up chances to meet  new people because of my  workout schedule | □ | □ | □ | □ | □ |

**EAT 26 - Eating Attitude Test 26**

Garner, D.M., Olmsted, M.P., Bohr, Y., and Garfinkel, P.E. (1982). The Eating Attitudes Test: Psychometric features and clinical correlates. Psychological Medicine, 12, 871-878

|  | **Always** | **Usually** | **Often** | **Sometimes** | **Rarely** | **Never** |
| --- | --- | --- | --- | --- | --- | --- |
| 1. Am terrified about being overweight | □ | □ | □ | □ | □ | □ |
| 2. Avoid eating when I am hungry | □ | □ | □ | □ | □ | □ |
| 3. Find myself preoccupied with food | □ | □ | □ | □ | □ | □ |
| 4. Have gone on eating binges where I feel that I may not be able to stop | □ | □ | □ | □ | □ | □ |
| 5. Cut my food into small pieces | □ | □ | □ | □ | □ | □ |
| 6. Aware of the calorie content of foods that I eat | □ | □ | □ | □ | □ | □ |
| 7. Particularly avoid food with a high carbohydrate content (i.e. bread, rice, potatoes,etc.) | □ | □ | □ | □ | □ | □ |
| 8. Feel that others would prefer if I ate more | □ | □ | □ | □ | □ | □ |
| 9. Vomit after I have eaten | □ | □ | □ | □ | □ | □ |
| 10. Feel extremely guilty after eating | □ | □ | □ | □ | □ | □ |
| 11. Am preoccupied with a desire to be thinner | □ | □ | □ | □ | □ | □ |
| 12. Think about burning up calories when I exercise | □ | □ | □ | □ | □ | □ |
| 13. Other people think that I am too thin | □ | □ | □ | □ | □ | □ |
| 14. Am preoccupied with the thought of having fat on my body | □ | □ | □ | □ | □ | □ |
| 15. Take longer than others to eat my meals | □ | □ | □ | □ | □ | □ |
| 16. Avoid foods with sugar in them | □ | □ | □ | □ | □ | □ |
| 17. Eat diet foods | □ | □ | □ | □ | □ | □ |
| 18. Feel that food controls my life | □ | □ | □ | □ | □ | □ |
| 19. Display self-control around food | □ | □ | □ | □ | □ | □ |
| 20. Feel that others pressure me to eat | □ | □ | □ | □ | □ | □ |
| 21. Give too much time and thought to food. | □ | □ | □ | □ | □ | □ |
| 22. Feel uncomfortable after eating sweets | □ | □ | □ | □ | □ | □ |
| 23. Like my stomach to be empty | □ | □ | □ | □ | □ | □ |
| 24. Engage in dieting behavior | □ | □ | □ | □ | □ | □ |
| 25. Enjoy trying new rich foods | □ | □ | □ | □ | □ | □ |
| 26. Have the impulse to vomit after meals | □ | □ | □ | □ | □ | □ |
